# A comparative analysis of infection prevention and control guidelines across Kenya, Tanzania, and Nigeria

**DOI:** 10.1017/ash.2026.10350

**Published:** 2026-05-08

**Authors:** Sheba M. Riziki, Muhydeen O. Olojo, Courage Chandipwisa, Zachariya Esther, Muhsin R. Athumani, Hafeez Hamza, Charles C. Ogu, Paul M. Iziomo, Ayodele O. Majekodunmi, Estelle Mbadiwe

**Affiliations:** 1 Kilifi District Hospital, Kenya; 2 Ducit Blue Foundation, Nigeria; 3 Pan African University Institute of Life and Earth Sciences, Ibadan, Nigeria; 4 University of Abuja, Nigeria; 5 Ministry of Health, United Republic of Tanzania; 6 Ajisefini Consulting, Nigeria

## Abstract

**Objective:**

Antimicrobial resistance (AMR) and healthcare-associated infections pose serious public health threats, particularly in Africa with limited health resources. Weak infection prevention and control (IPC) implementation worsens these challenges. To address this, Africa Centre for Disease Control (CDC) developed the IPC legal framework. This study compares Kenya, Nigeria, and Tanzania’s IPC guidelines with the framework and evaluates their alignment.

**Methods:**

This policy analysis compares the IPC guidelines of three countries and assesses their alignment against the attributes of the Africa CDC Legal IPC Framework. Scores were assigned based on the level of alignment with the attributes, aggregated into percentages, and compared across countries. This study was developed under the 2023–24 cohort 3, of Ducit Blue Foundation’s pioneering and award-winning AMR One Health Pan-African Internship/Mentorship Program.

**Results:**

Facility-level IPC budgeting was included in Nigeria and Tanzania’s guidelines but absent in Kenya. Training responsibilities differed, with Kenya and Tanzania authorizing national and regional bodies, while Nigeria assigned this solely to facility IPC teams. Overall alignment of the IPC guidelines with the Africa CDC Legal Framework was 69%. Tanzania had 76% alignment, while Kenya and Nigeria recorded 65% each. There were no significant variations in alignment between countries with the benchmark framework (X^2^ = 3.75, *p*-value = .153).

**Conclusion:**

This study represents the first attempt at a detailed policy analysis of IPC guidelines across Africa against a backdrop of the relevant regional instruments. The countries show good IPC alignment with the benchmark instrument, reflecting both regional commitment and local adaptation.

## Introduction

Healthcare-associated infections (HAIs), including antimicrobial-resistant HAIs (HAIs–AMR) occur during healthcare delivery or after patient discharge.^1^ They endanger patients and healthcare workers (HCWs) by spreading resistant pathogens and reducing treatment effectiveness.^2,3^ HAIs are among the most common adverse health events, with multidrug-resistant organisms accounting for a large share, imposing substantial clinical and economic burdens on health systems.^4^

Globally, HAIs pose a major health burden. Around one-quarter of hospital-treated sepsis cases and 48.7% of sepsis with organ dysfunction in adult ICUs are healthcare-associated. About 7% of patients in acute-care hospitals in high-income countries and 15% in low-and middle-income countries acquire at least one HAI, reaching 30% in intensive care units.^5^ HAIs and HAIs-AMR prolong hospital stay, cause complications, disability, and premature death, while imposing heavy social and economic costs on societies.^6,7^ For health systems, they raise care demands and costs.^8^ HAI-associated sepsis causes death in ∼ 25% of cases, rising to 50% in ICUs.^7^ Without effective prevention, HAIs could cause 3.5 million deaths annually by 2,050.^9^

Infection prevention and control (IPC) is an evidence-based strategy to limit pathogen transmission, reduce HAIs, and combat AMR.^10,11^ Effective IPC requires coordinated action across government, facilities, HCWs, patients, and visitors.^11^ IPC interventions like hand hygiene, surgical site infection prevention, injection safety, and AMR containment are central to quality healthcare.^12^

To support IPC implementation, WHO issued the Core Components Guidelines in 2016 for national and facility-level systems.^12^ Yet implementation gaps remain. By 2024, 9% of countries lacked national IPC plan and only 39% had IPC programs fully implemented.^9^ Of 150 countries assessed, only nine met all minimum requirements and 21 achieved ≥90% compliance.^9^ In Africa, persistent gaps in financing, training evaluation, surveillance, and monitoring led to the lowest global average IPC capacity in the 2023 SPAR assessment.^9,13^

Africa has established several guidelines to help countries and health facilities (HFs) combat HAIs and AMR.^14^ Yet IPC implementation varies due to local contexts and resource constraints.^15^ To address recurring outbreaks and persistent challenges, the Africa Centre for Disease Control (CDC) developed the IPC Legal Framework in 2022 as a regional benchmark to strengthen governance, accountability, surveillance, and resourcing while allowing country-specific adaptation. However, extent of its translation into national policies remains unclear. Misalignment between regional guidance and national policy risks systemic gaps that undermine implementation, comparability, and coordinated responses to HAIs and HAIs-AMR.

Despite recent disease outbreaks in Africa, evidence on regional alignment and combination of IPC interventions remains limited. No prior study has comprehensively assessed African IPC guidelines against the Africa CDC Legal IPC Framework. Existing research focused on national program status, facility performance, and country-specific regulations. Therefore, regional assessments of guideline alignment using a standardized framework are needed.

This study compares the national IPC guidelines of Kenya, Nigeria, and Tanzania and assesses alignment with the Africa CDC IPC Legal Framework to identify gaps, inconsistencies, and opportunities for regional harmonization. Findings from this study will inform future revisions of IPC guidelines and guide targeted support from Africa CDC and partners to strengthen IPC across Africa.

### Africa CDC IPC legal framework (2022)

Following the Ebola and COVID-19 outbreaks, the role of hospitals in disease transmission became clearer, especially the impact of HAIs on strained health systems. Strengthening IPC became imperative. In 2017, Africa CDC launched its AMR control framework with strong IPC emphasis,^16,17^ followed by 2018 consultations to prioritize implementation and set minimum standards for safe facilities. In 2019, further consultations with WHO and AU Member States produced detailed IPC requirements and a proposed public health law on accountability, resourcing, leadership, and monitoring.

Subsequently, the IPC Legal Framework^13^ was endorsed by AU in July 2022 during its 41st Executive Council session in Lusaka, Zambia.^18^ Structured into six domains aligned with WHO core components, the framework sets minimum expectations for governance, surveillance, implementation, and accountability, while allowing country-specific adaptation, hence a suitable tool for comparative policy analysis.

## Methods

### Study setting

This study emerged from the third cohort of Ducit Blue Foundation (DBF) award-winning AMR One Health Pan-African Internship/Mentorship Programme, delivered with One Health Lessons and Nigerian Institute of Medical Research. The program strengthens policy analysis and evidence translation among early-career public health professionals in Africa, building capacity for succession planning and ensuring the next generation understands AMR governance, to develop NAPs and IPC strategies informed by local evidence.

### Study area

This study reviewed IPC guidelines from Kenya, Tanzania and Nigeria, purposively selected based on interns’ countries of origin and document availability.

### Study design

This study used a two-phase approach. Phase One compared national IPC guidelines to examine strategies, and Phase Two analyzed their alignment with the Africa CDC IPC Legal Framework to assess regional compliance.

### Data extraction

The documents were sourced from publicly available, open-access online platforms and include:

- Africa CDC IPC Legal Framework, 2022.
- Kenya National Infection Prevention and Control Strategic Plan for Health Care Services 2021–2025
- The Nigerian Manual of Infection Prevention and Control, 2021.
- National Infection Prevention and Control Guidelines for Health Care Services in Tanzania, 2018.

### Framework

The Africa CDC IPC Legal Framework includes six domains with defined legal and policy attributes. In this study, full alignment was noted when national IPC guidelines addressed all core attributes within a domain.

**Domain 1 (Establishment of a National IPC Programme)**: include a national program with an allocated budget, designated sub-national units for implementation, and authority to coordinate IPC activities with ministries, agencies, professional societies, and institutions.

**Domain 2 (Development and adoption of evidence-based guidelines)**: covers adoption of national IPC standards, their review and revision at intervals not exceeding five years, coherence among legal instruments for oversight and approval, and implementation across all administrative levels.

**Domain 3 (Education and training):** covers authority to develop and periodically revise IPC curricula, coordination with relevant ministries or agencies, facility-level responsibility for training healthcare personnel, and annual IPC training.

**Domain 4 (Incorporating HAIs into surveillance)**: covers authorization of a multidisciplinary national system for HAIs and HAI-related AMR, facility-level surveillance requirements, funding for compliance, coordination with national initiatives, and protection of health data.

**Domain 5** (**Monitoring, audit, and feedback of IPC compliance**): covers authorization to create a system that monitors compliance with national IPC standards, including feedback to facilities, data validation, analysis and reporting to guide the National IPC Programme, and provisions for remediation, accountability, and investigation of negligence.

**Domain 6 (Facility-level IPC programs):** authorizes healthcare facilities to establish programs with dedicated budgets, develop SOPs consistent with national and international standards, ensure adequate staffing of trained IPC personnel, and comply with facility design and infrastructure regulations set by the relevant authority.

### Data analysis

Data was analyzed with Microsoft Excel 2019 and AntConc 4.3.1, a freeware corpus analysis toolkit developed in 2002 by Laurence Anthony at Waseda University, Tokyo, Japan. AntConc supports textual and corpus-based qualitative analysis. Keywords and codes were developed around the benchmark framework domains and reviewed by two AMR experts for reliability. IPC guideline documents were imported into AntConc and analyzed with concordance and keyword search functions to identify predefined terms, including references outside designated sections. Outputs were manually reviewed for contextual accuracy. Extracted IPC actions were compared against the Africa CDC IPC Legal Framework and assessed for alignment by scope of coverage using a three-point traffic-light scale^18^:

- 1 = full alignment (green)
- .5 = partial alignment (yellow)
- 0 = no alignment (red)

Scores were aggregated and converted to percentages to evaluate overall alignment across countries. Statistical associations were tested with χ^2^, significance set at *P* < .05. Results were presented with thematic maps and column charts.

## Results

### Comparison of the IPC guidelines

The IPC guidelines of the countries showed varying similarities and differences across the domains of the Africa CDC IPC Legal Framework.

### National IPC program

Nigeria and Kenya explicitly established national IPC programs aligned with the benchmark framework. Kenya mandated oversight at both central and peripheral levels, while Nigeria limited regulatory responsibility mainly to facilities. In contrast, Tanzania did not establish a national IPC program, assigning IPC duties instead to the Ministry of Health (MoH), Community Development, Gender, Elderly, and Children (MOHCDGEC).

### National standards for IPC

All three countries mandated national IPC standards. Kenya and Tanzania authorized enforcement across all administrative levels, while Nigeria restricted implementation to facilities. Nigeria alone required annual review, making it the only guideline with a defined review time line.

### Education and training of HCWs and IPC professionals

All three countries mandated IPC education and training for HCWs. Kenya assigned responsibility to multiple stakeholders, including national ministries, county departments, partners, and hospital leadership. Tanzania designated the MOHCDGEC and the President’s Office—Regional Administration and Local Government (PORALG), while Nigeria relied on facility-level IPC committees. Training frequency differed: Kenya every three months, Tanzania every six, and Nigeria annually.

### National system of surveillance for HAIs and HAIs-AMR

Nigeria and Tanzania authorized national surveillance systems for HAIs and HAIs–AMR. Nigeria assigned oversight to the Nigeria Centre for Disease Control, while Tanzania designated PORALG and the Quality Improvement Team (QIT). Kenya did not establish a nationwide system but mandated strengthening surveillance and notification for HAIs, AMR, and hospital outbreaks within healthcare facilities.

### National system for monitoring compliance with national IPC standards at facility-level

All three countries established facility-level systems to monitor compliance with national IPC standards. Kenya authorized an audit tool for assessing interventions across all healthcare levels, Nigeria assigned monitoring to IPC teams or focal persons, and Tanzania designated the QIT and Work Improvement Team.

### Facility-level IPC

All three countries authorized IPC programs within HFs. Unlike Nigeria and Tanzania, Kenya’s guideline does not specify a dedicated budget or define the number and cadre of trained IPC personnel.

### Overall alignment with Africa CDC IPC legal framework

**Figure 1.**
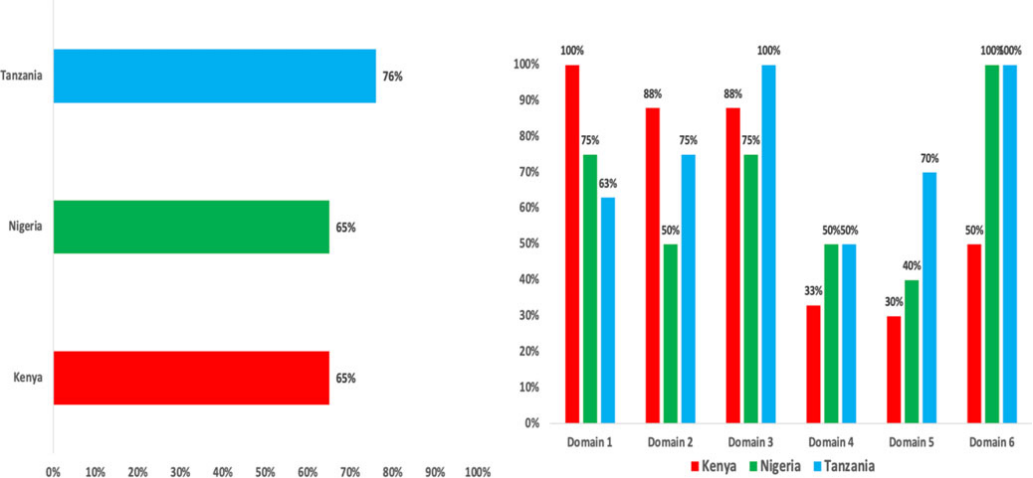
(Left): Alignment of national IPC guidelines with the Africa CDC IPC legal framework; (Right): Alignment of selected countries’ IPC guidelines with the domains of the Africa CDC IPC legal framework.

The average overall alignment of the three countries’ IPC guidelines with the Africa CDC Legal Framework was 69%, with Tanzania demonstrating the highest alignment at 76% (Figure 1). There was no statistically significant difference in overall alignment among the countries (X^2^ = 3.75, *P* = .153).

### Countries’ IPC guidelines alignment with domains of Africa CDC IPC legal framework

For Domain 1 (establishing a national IPC program), Kenya’s IPC guideline achieved full alignment (100%), compared with 75% for Nigeria and 63% for Tanzania. In Domain 2, Kenya also demonstrated the highest alignment (88%), followed by Tanzania (75%) and Nigeria (50%) (Figure 1).

### Alignment with specific attributes of domains of Africa CDC IPC legal framework

#### Domain 1

The countries demonstrated strong overall alignment (79%) with domain 1. Complete alignment (100%) was observed in the identification of sub national administrative units, indicating that decentralization of responsibilities is a common strength. Also, the NAPs demonstrated consistent strength in the establishment of national IPC programs and associated budgets, where alignment reached 83%, indicating that financial and structural provisions are relatively well established. In contrast, collaboration with other relevant authorities was only partially aligned (50%), pointing to gaps in cross sectoral engagement (Table 1).

**Table 1.**
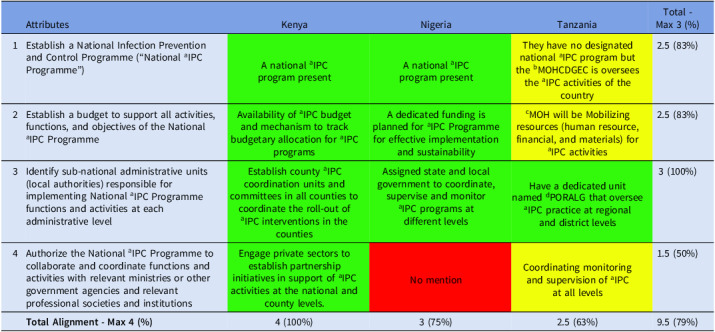
Alignment of the countries’ IPC guidelines with domain 1 of Africa CDC

*Key—green (complete alignment—2), yellow (partial alignment—1), and red (no alignment—0). ^a^infection Prevention and Control; ^b^Ministry of Health, Community Development, Gender, Elderly, and Children; ^c^Ministry of Health; ^d^President’s Office, Regional Administration and Local Government.

#### Domain 2

All three countries fully aligned (100%) with adopting national IPC standards, showing strong commitment to a unified framework. Regular review of these standards was only partially aligned (50%), revealing limited mechanisms for updating guidance. Strong alignment (83%) was observed for both integration of legal instruments and implementation across administrative levels, indicating that while structures are largely in place, the main weakness lies in keeping standards current (Table 2).

**Table 2.**
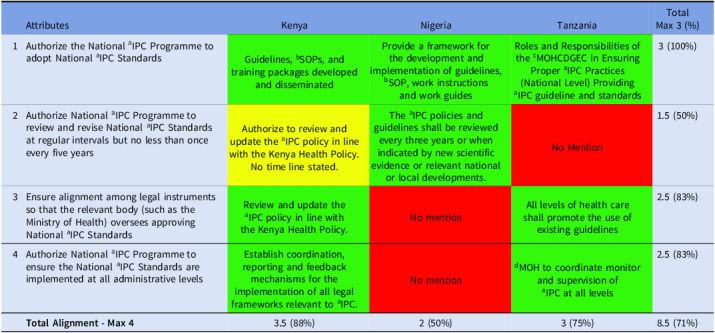
Alignment of the countries’ IPC guidelines with domain 2 of Africa CDC IPC legal framework

*Key—green (complete alignment—2), yellow (partial alignment—1), and red (no alignment—0). ^a^infection Prevention and Control; ^b^Standard Operating Procedure; ^c^Ministry of Health, Community Development, Gender, Elderly, and Children; ^d^Ministry of Health.

#### Domain 3

All three countries fully aligned (100%) with developing an IPC training curriculum and designating training responsibilities within HFs, reflecting strong institutional commitment to capacity building. They also showed strong alignment (83%) with the requirement for annual IPC training, suggesting that while training is widely established, it is not yet consistently implemented across all contexts (Table 3).

**Table 3.**
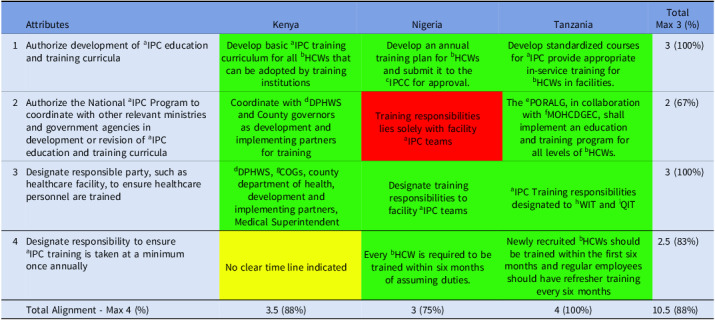
Alignment of the countries’ IPC guidelines with domain 3 of Africa CDC IPC legal framework

*Key—green (complete alignment—2), yellow (partial alignment—1), and red (no alignment—0). ^a^infection Prevention and Control; ^b^Health Care Workers; ^c^Infection Prevention Control Committee; ^d^Division of Patient and Health Worker Safety; ^e^President’s Office, Regional Administration and Local Government; ^f^Ministry of Health, Community Development, Gender, Elderly, and Children; ^g^Council of Governors; ^h^Work Improvement Team; ^i^Quality Improvement Team.

#### Domain 4

This domain assessed national surveillance systems for HAIs and HAI-AMR across all countries. Full alignment (100%) was observed in authorizing surveillance system, reflecting strong surveillance policy commitment across. Moderate alignment (67%) was observed for establishing surveillance programs and facility level requirements, indicating consistent efforts across board. Coordination with national initiatives was weaker (33%), and no alignment was found on funding facility compliance or protecting health data, highlighting critical gaps in collaboration, financing, and data governance (Table 4).

**Table 4.**
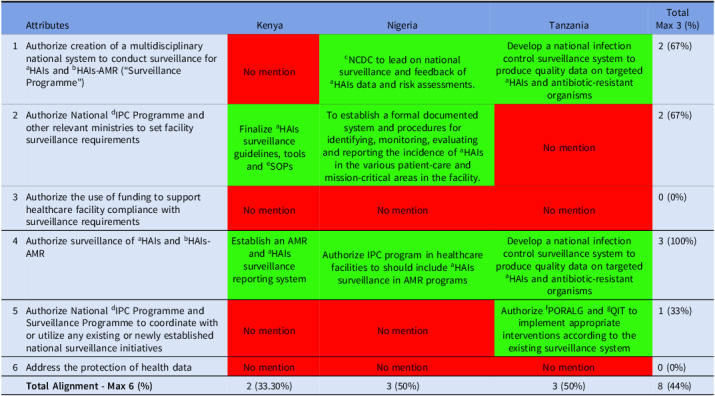
Alignment of the countries’ IPC guidelines with domain 4 of Africa CDC IPC legal framework

*Key—green (complete alignment—2), yellow (partial alignment—1), and red (no alignment—0). ^a^Healthcare-associated infections; ^b^Healthcare-associated antimicrobial resistance; ^c^Nigeria Centre for Disease Control and Prevention; ^d^infection Prevention and Control; ^e^Standard Operating Procedure; ^f^President’s Office, Regional Administration and Local Government; ^g^Quality Improvement Team.

#### Domain 5

All three countries fully aligned (100%) with establishing systems to monitor compliance with national IPC standards but partially align (50%) with activities to monitor and provide feedback, indicating strong commitment to oversight but weak implementation. Low alignment (17%) was observed for independent auditing of IPC standards, while none demonstrated mechanisms to remedy non-compliance, highlighting major gaps in enforcement and accountability (Table 5).

**Table 5.**
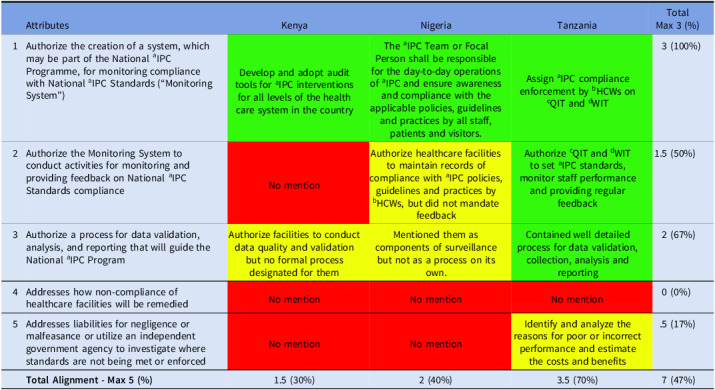
Alignment of the countries’ IPC guidelines with domain 5 of Africa CDC IPC legal framework

*Key—green (complete alignment—2), yellow (partial alignment—1), and red (no alignment—0). ^a^infection Prevention and Control; ^b^Health Care Workers; ^c^Quality Improvement Team; ^d^Work Improvement Team.

#### Domain 6

A strong facility-based IPC commitment was observed across the countries. Complete alignment (100%) was achieved in establishing facility-based IPC programs and issuing regulations for HFs. High alignment (83%) was observed for developing facility-level SOPs consistent with national and international standards, while moderate alignment (67%) was seen for budgeting and staffing, pointing to less consistency in resource allocation (Table 6).

**Table 6.**
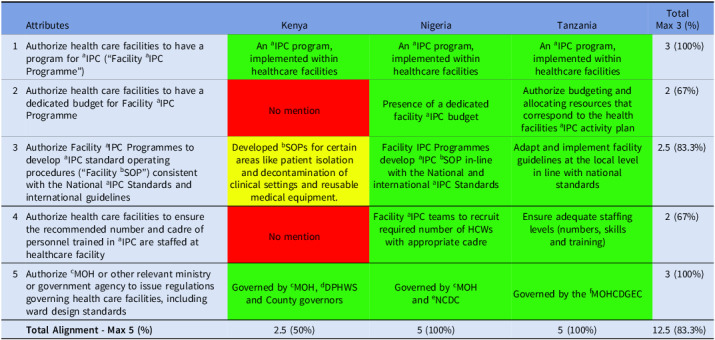
Alignment of the countries’ IPC guidelines with domain 6 of Africa CDC IPC legal framework

*Key—green (complete alignment—2), yellow (partial alignment—1), and red (no alignment—0). ^a^infection Prevention and Control; ^b^Standard Operating Procedure; ^c^Ministry of Health; ^d^Division of Patient and Health Worker Safety; ^e^Nigeria Center for Disease Control and Prevention; ^f^Ministry of Health, Community Development, Gender, Elderly, and Children.

## Discussion

This study analyzed IPC guidelines from Kenya, Nigeria, and Tanzania to compare national strategies and assess compliance with the Africa CDC IPC Legal Framework. The framework serves as a benchmark for strengthening IPC programs and guiding policy where legal instruments are limited.^13^ By balancing local adaptation with regional consistency, alignment assessment clarifies how national policies reflect both regional standards and country-specific needs.

### Alignment with Africa CDC IPC legal framework

HAIs burden healthcare systems by increasing costs, morbidity, mortality, hospital stays, and reducing quality of life.^19^ In Africa and other low-resource settings, systemic challenges intensify this impact.^20^ The relatively strong alignment across IPC guidelines (average score 69%) reflects shared recognition of the threat posed by HAIs and HAIs–AMR. Aligning with regional standards is critical, as evidence shows effective IPC measures can cut HAIs by up to 50%.^21–23^ This alignment signals readiness to adopt interventions that lower infection rates, reduce hospital stays and costs, and improve patient outcomes.

### Countries’ guidelines alignment with domains of IPC legal framework

Africa CDC IPC Legal framework highlights the need for national IPC programs.^12^ Kenya and Nigeria have dedicated programs led by the MoH and NCDC, while Tanzania assigns oversight to a broader agency (MOHCDGEC). Dedicated programs strengthen accountability and coordination, whereas broader agencies risk competing priorities and implementation gaps.^24,25^ Stronger accountability structures increase the likelihood of consistent IPC policy enforcement, reducing preventable infections.

All three countries have established national IPC standards, although differences in alignment were observed across this domain. Nigeria’s guideline uniquely mandates annual review of IPC standards, highlighting the importance of regular updates to reflect evolving IPC practices.^26^ Kenya and Tanzania extend IPC standards across all administrative levels, whereas Nigeria’s focus is largely limited to HFs. While facility-level implementation remains essential, broader administrative coverage may support improved disease awareness and hygiene practices beyond hospital settings. Regular updates and wider administrative coverage ensure that IPC standards remain current and widely applied, which is essential for maintaining effectiveness in rapidly changing health contexts.

Strong alignment was observed across all three countries in IPC education and training for HCWs, consistent with evidence that training reduces HAIs and HAIs–AMR.^26,27^ The Africa CDC framework stresses collaboration with relevant agencies, fulfilled by Kenya and Tanzania. Emerging evidence highlights integrating IPC training with bioinformatics, IT, and data analysis to match growing digitalization.^26^ High alignment in training is critical, as well-trained HCWs are frontline implementers, and without consistent training, even robust national IPC standards risk poor translation into practice.

Alignment on surveillance systems for HAIs varied. Nigeria and Tanzania outlined national systems coordinated by central bodies, while Kenya focused on facility-level surveillance. This reflects resource and capacity differences, as nationwide systems require major investment and many countries rely on facility monitoring.^28^ Yet HAIs can spread beyond facilities via HCWs and visitors, making broader coverage vital.^29^ Localized surveillance limits early outbreak detection and response, allowing HAIs to spread unchecked across communities and undermining public health protection.

Differences were noted in monitoring compliance with national IPC standards. Tanzania showed strong alignment by mandating direct facility feedback to the national IPC program, while Kenya relied on indirect mechanisms such as newsletters, and Nigeria required facilities mainly to keep records. Effective monitoring depends on clear feedback loops to ensure accountability. None of the guidelines addressed mechanisms for managing non-compliance, negligence, or malfeasance—likely reflecting workforce constraints where punitive measures could worsen shortages.^30^ Still, defined compliance mechanisms are vital. Without them, IPC standards risk remaining aspirational rather than operational, weakening patient safety and public health outcomes.

WHO stresses the need for facility-level IPC programs,^12^ a requirement met by all three countries. Unlike Nigeria and Tanzania, Kenya’s guideline does not allocate a dedicated budget or specify staffing levels. Clear funding and staffing policies are vital, as facility IPC teams are the frontline of implementation. Without adequate resources, teams struggle to sustain practices, limiting their ability to prevent infections at the point of care.

We acknowledged several limitations in this study. First, it relied solely on published documents, which may not reflect actual practices or informal procedures. Second, interpretation of policy language involved some subjectivity, and terminology variations may have affected coding consistency. Finally, AU frameworks provide broad continental guidance, while each country retains authority to set priorities and adapt policies to its context. Thus, alignment with regional frameworks may not fully capture national decision-making, and differing priorities could limit comparability across countries.

In conclusion, the countries showed moderate-to-strong alignment with the Africa CDC IPC Legal Framework. Strongest alignment was in education and training and facility-level IPC programs, while weaker alignment appeared in HAIs surveillance and monitoring compliance. These findings highlight that workforce capacity and accountability are critical for implementation. Structural alignment provides a foundation, but without enforcement and trained staff, the public health impact of IPC measures is greatly reduced.

This study forms part of the succession planning within the DBF’s One Health Pan-African AMR Internship/Mentorship Programme. It reflects broader efforts to strengthen policy analysis and evidence translation capacity among early-career public health professionals in Africa. Such capacity building is critical for succession planning in Africa, ensuring that tomorrow’s public health workforce have a good grasp of AMR governance, to support the development of effective NAPs, and ensure that IPC strategies are informed by locally generated evidence.

## Data Availability

Data supporting this study are included within the article and/or supporting materials.
